# Liposomal Cancer Drug Database (LCDD): a comprehensive resource for liposome research in cancer therapy and diagnosis

**DOI:** 10.1093/database/baaf042

**Published:** 2025-10-31

**Authors:** Marziyeh Mousazadeh, Babak Khorsand, Sayed Mostafa Modarres Mousavi, Hooman Mahmoudi Aznaveh, Mohamad Hassan Fouani, Faezeh Mousazadeh, Mehrnaz Rad-Faraji, Arezoo Golestanipour, Soheila Mohammadi, Fateme Zarein, Alireza Dantism, Javad Zahiri, Maryam Nikkhah

**Affiliations:** Department of Nanobiotechnology, Faculty of Biological Sciences, Tarbiat Modares University, Jalal Al Ahmad Highway, Tehran, 14115-154, Iran; Department of Neurology, University of California, Manchester Avenue, Irvine, CA 92617, United States; Department of Nanobiotechnology, Faculty of Biological Sciences, Tarbiat Modares University, Jalal Al Ahmad Highway, Tehran, 14115-154, Iran; Department of Nanobiotechnology, Faculty of Biological Sciences, Tarbiat Modares University, Jalal Al Ahmad Highway, Tehran, 14115-154, Iran; Department of Nanobiotechnology, Faculty of Biological Sciences, Tarbiat Modares University, Jalal Al Ahmad Highway, Tehran, 14115-154, Iran; Department of Nanobiotechnology, Faculty of Biological Sciences, Tarbiat Modares University, Jalal Al Ahmad Highway, Tehran, 14115-154, Iran; Department of Nanobiotechnology, Faculty of Biological Sciences, Tarbiat Modares University, Jalal Al Ahmad Highway, Tehran, 14115-154, Iran; Department of Plant Biotechnology, Faculty of Agricultural Sciences, University of Guilan, Persian Gulf Highway, Rasht, 41996-13776, Iran; Nano Drug Delivery Research Center, Health Technology Institute, Kermanshah University of Medical Sciences, Abrisham Avenue, Kermanshah, 6714415153, Iran; Department of Nanobiotechnology, Faculty of Biological Sciences, Tarbiat Modares University, Jalal Al Ahmad Highway, Tehran, 14115-154, Iran; Department of Biophysics, Faculty of Biological Sciences, Tarbiat Modares University, Jalal Al Ahmad Highway, Tehran, 14115-154, Iran; Autism Center of Excellence, Department of Neurosciences, University of California, San Diego, 9500 Gilman, La Jolla, CA 92037, United States; Department of Nanobiotechnology, Faculty of Biological Sciences, Tarbiat Modares University, Jalal Al Ahmad Highway, Tehran, 14115-154, Iran

## Abstract

The Liposomal Cancer Drug Database (LCDD) is a comprehensive web-based resource that provides an extensive overview of liposome-based formulations used in cancer therapy and diagnostics. LCDD compiles data from about 4500 research articles, categorizing information into 14 key attributes for each research article such as type of cancer, liposome size, type of experiments, cell line type, *in vivo* animals, liposome function, and type of cargo. The database is built on direct literature investigations and serves as a critical tool for researchers developing liposome-based cancer therapeutic drugs, diagnostic platforms, or theragnostic solutions. LCDD is available for noncommercial purposes as a free database at https://bioinformaticscollege.ir/lcdd/.

## Introduction

### Cancer

Cancer, the second major cause of death worldwide, claimed the lives of ten million people in 2020 rendering it a serious global health problem. Almost for all malignancies, the likelihood of survival increases dramatically if the malignancy is diagnosed at its early stages and treated promptly [1]. The lack of effective therapeutic and early diagnostic tools are the main causes of cancers poor prognosis [2]. Cancer treatment includes surgery, chemotherapy, or radiotherapy, either alone or in combination. Despite noticeable clinical improvements, these therapeutic methods still suffer from drawbacks such as severe side effects, systemic toxicity, nonspecificity, and lack of effectiveness [3]. Furthermore, traditional cancer detection and imaging techniques are time-consuming, may not reliably distinguish between benign and malignant tumours, and cannot diagnose cancer at pre-early stages [4].

Nanoparticle-based technologies have been developed as promising cancer therapeutic and diagnostic tools that can be used for cancer drugs personalization and more effective tumour suppression [5]. Features such as high surface area-to-volume ratio and increased reactivity of nanoparticles contribute to their profound therapeutic and diagnostic efficiency. Enhanced permeability and retention effect, which results from high vascular permeability and poor lymphatic drainage, enables passive targeting and accumulation of nanoparticles (NPs) harbouring therapeutic and diagnostic agents at the tumour site [6]. High surface area-to-volume ratio of NPs enables their efficient functionalization with various targeting moieties such as antibodies, peptides, aptamers, and carbohydrates [7]. Functionalization of NPs elevates their payload at tumour site, enhancing specificity and sensitivity as well as diminishes side effects of the therapeutic agents [8].

### Liposomes

Liposomes, discovered in 1964, have gained wide attention for drug delivery, targeting, solubilization, stabilization, pharmacokinetic improvement, and as diagnostic tools [9]. Liposomes are prepared from phospholipids, either synthetic or natural, with a unique vesicular structure that helps entrap hydrophilic drugs in the core and lipophilic drugs between the phospholipid layers [10]. Liposomal drug delivery systems are capable of carrying large drug payloads, enhance bioavailability through drug protection against degradation and body’s immune system, and are tunable in their physicochemical properties [11]. Liposomes are biodegradable and are considered biocompatible [12]. Surface functionalization of liposomes with various moieties may increase their circulation half-life, facilitates target specificity, promotes cellular uptake, and facilitates drug release in response to stimuli [2, 13]. Several liposomal formulations have entered clinical studies. For instance, liposomal doxorubicin functionalized with antibodies targeting the epithelial growth factor receptor are one of these novel formulations with enhanced targeting capabilities. Some of the liposomal formulations available on the market for cancer therapy are Doxil (liposomal doxorubicin), Lipoplatin (liposomal cisplatin), DaunoXome (liposomal daunorubicin), Marqibo (liposomal vincristine), Onivyde (liposomal irinotecan), and Vyxeos (liposomal daunorubicin and cytarabine) [14, 15]. Several researches have been employed liposomes as delivery vehicles for diagnostic agents in fluorescence, nuclear magnetic resonance, and ultrasound based imaging platforms. Despite significant progress in cancer treatment and diagnosis through liposomal drug delivery technologies, several obstacles have limited the clinical use of liposomal formulations. To achieve the full therapeutic and diagnostic potential of liposomal formulations, tumour heterogeneity and biological hurdles must be overcome. Other barriers include cellular internalization and effective tumour tissue penetration [16]. Continuous advances in liposome research and its applications in cancer treatment and diagnosis have resulted in the accumulation of a vast amount of data over the past few decades. Herein, a comprehensive database that consolidates all relevant liposome research is reported. This database would make it easier for researchers to access and review existing studies, methodologies, and findings. This database facilitates collaboration among researchers by providing a shared platform where they can find comprehensive data, leading to more robust and innovative research outcomes. Researchers can save time by quickly finding relevant studies and avoiding duplication of efforts, allowing them to focus on advancing new ideas and experiments. The database allows for the analysis of trends and patterns in liposome research, helping to identify gaps in knowledge and potential areas for future investigation.

**Figure 1.**
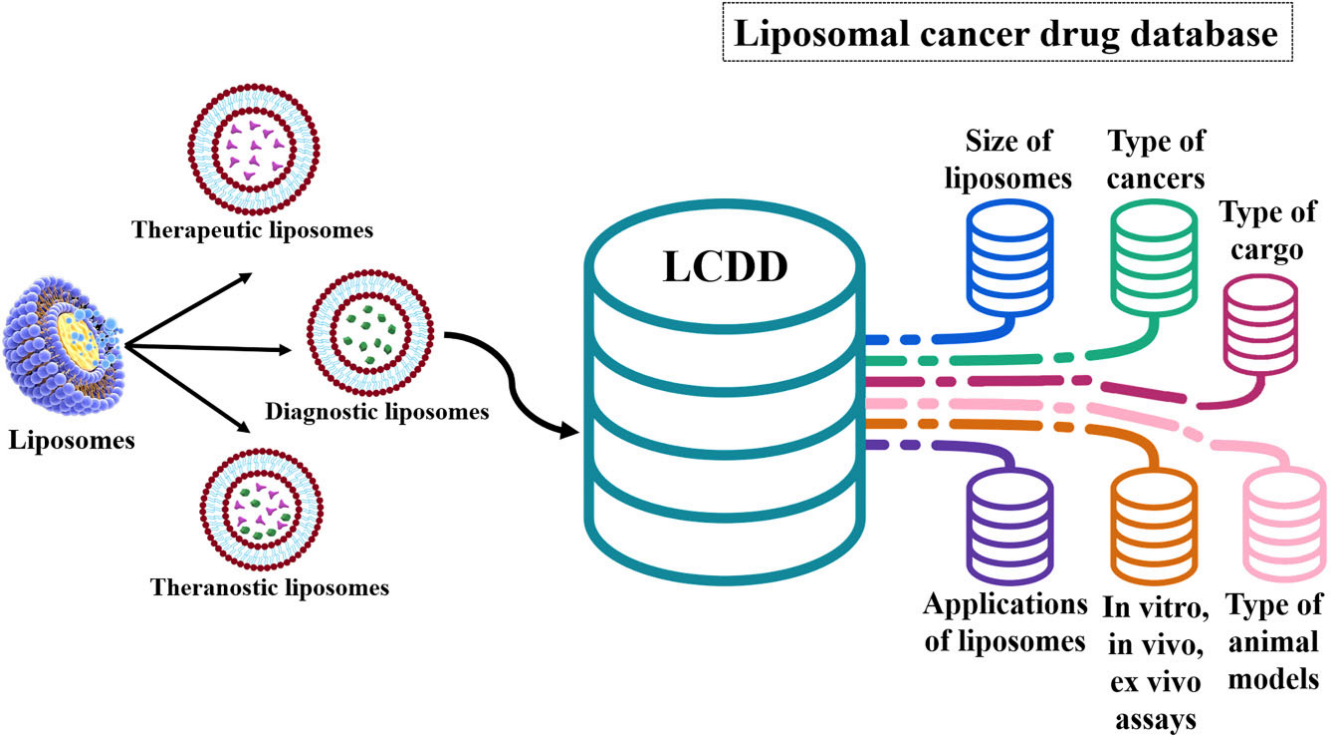
The data from liposome research articles were manually extracted and incorporated into a comprehensive database (LCDD).

Figure 1 shows the importance and attributes of LCDD.

### Database description

#### Data collection and validation

A vast array of information on various attributes of liposomal formulations in cancer therapy and diagnostics has been gathered, reviewed, and categorized in the current database. By searching various article databases such as Scopus, Web of Knowledge, PubMed, and Google Scholar, 8786 papers up to December 2024 were found using keywords: ‘liposome’, ‘cancer’, and ‘drug delivery’, as well as searching by various cancer names (e.g. breast, brain, lymphoma, etc.) and liposome anonyms (e.g. LNP). After excluding reviews, nonrelated, non-English articles, and those without available full text, data from the selected papers were extracted based on cancer type, liposome size (<100, 100–200, >200 nm), type of experiments (*in vitro, ex vivo, in vivo*, clinical trial), cell lines, animal models, function (therapeutic, diagnostic, theranostic), and type of cargos (chemical compound, nucleic acid, protein and peptide, nanoparticle, lipid, polysaccharide). Cell line names were standardized according to the ATCC (American Type Culture Collection) database to ensure accurate introduction and spelling.

To facilitate data management, a robust Content Management System using PHP for the backend and Vue.js (version 3) for the frontend were developed, with MySQL as the database management system for CRUD (Create, Read, Update, Delete) operations. In order to maintain data integrity, several validation mechanisms including duplicate entry prevention, mandatory field validation, data type validation, format validation, cross-field validation, and automated error reporting mechanisms were implemented.

Individuals extracting data were trained to follow a standardized procedure, ensuring consistency. After every 100 articles, extracted data underwent random checks by two independent reviewers. At the end of the data preparation, data were randomly verified twice against the original articles to ensure accuracy.

#### Database implementation details

The LCDD database has been implemented with a robust backend using PHP and a MySQL database. The choice of PHP and MySQL provides a reliable and efficient foundation for storing, managing, and retrieving data. To enhance the user experience, a responsive version of the database has been implemented using Bootstrap, ensuring that users can seamlessly access and interact with the database across various devices and screen sizes.

HTML and CSS work hand in hand to structure and style the LCDD database’s user interface. HTML defines the content and structure, while CSS brings life to the interface with custom styling. Together, they create an intuitive and visually appealing environment for users to interact with the database and access its valuable data.

To improve the interactivity and speed of data retrieval, jQuery has been utilized to fetch data from the database without requiring page refreshes. This AJAX-based approach enables a smoother user experience by dynamically updating content on the page.

To facilitate efficient data filtering and search capabilities, selectize.js and a Table sort library have been integrated into the database. These powerful libraries allow users to easily filter and sort data within the Tables, enabling them to quickly find the information they need.

Additionally, data retrieval in JSON format and the conversion of data into CSV files have been implemented. The ability to convert the retrieved data into CSV files provides users with the flexibility to analyse and manipulate the data using external tools and software.

Overall, the LCDD database implementation leverages PHP and MySQL as the backend technologies, while incorporating Bootstrap for responsiveness, jQuery for dynamic data retrieval, selectize.js and Table sort library for efficient data filtering, and JSON to CSV conversion for data flexibility. These components work together to create a robust and user-friendly environment for accessing and analysing the database’s data.

#### Web interface

LCDD is a free and web-based online database provided at https://bioinformaticscollege.ir/lcdd/.

**Figure 2.**
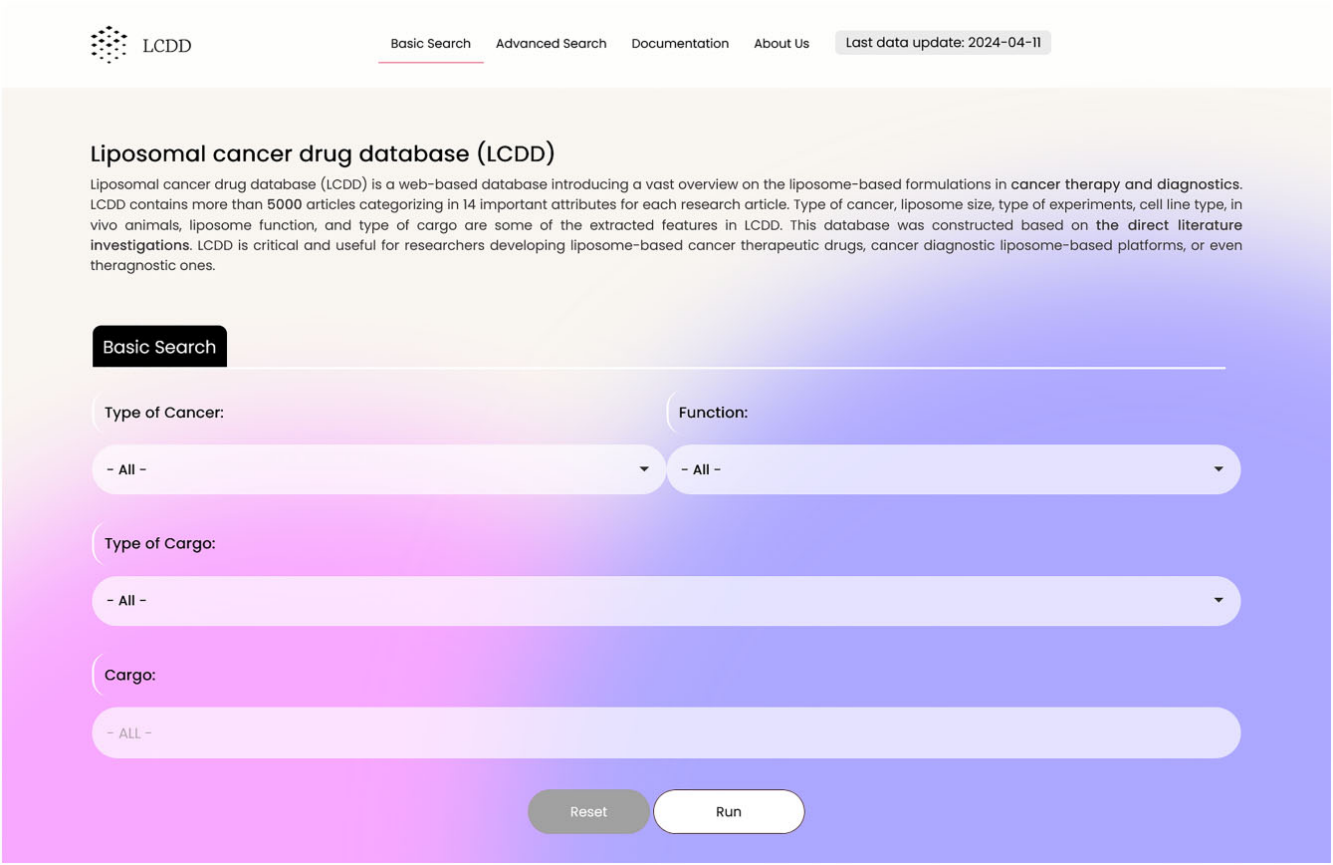
LCDD interface—basic search.

To provide a user-friendly graphical interface, multibutton-based search features are available, displaying results in a categorized order. A snapshot of the LCDD GUI (basic search) is presented in Fig. 2.

#### Search section

**Figure 3.**
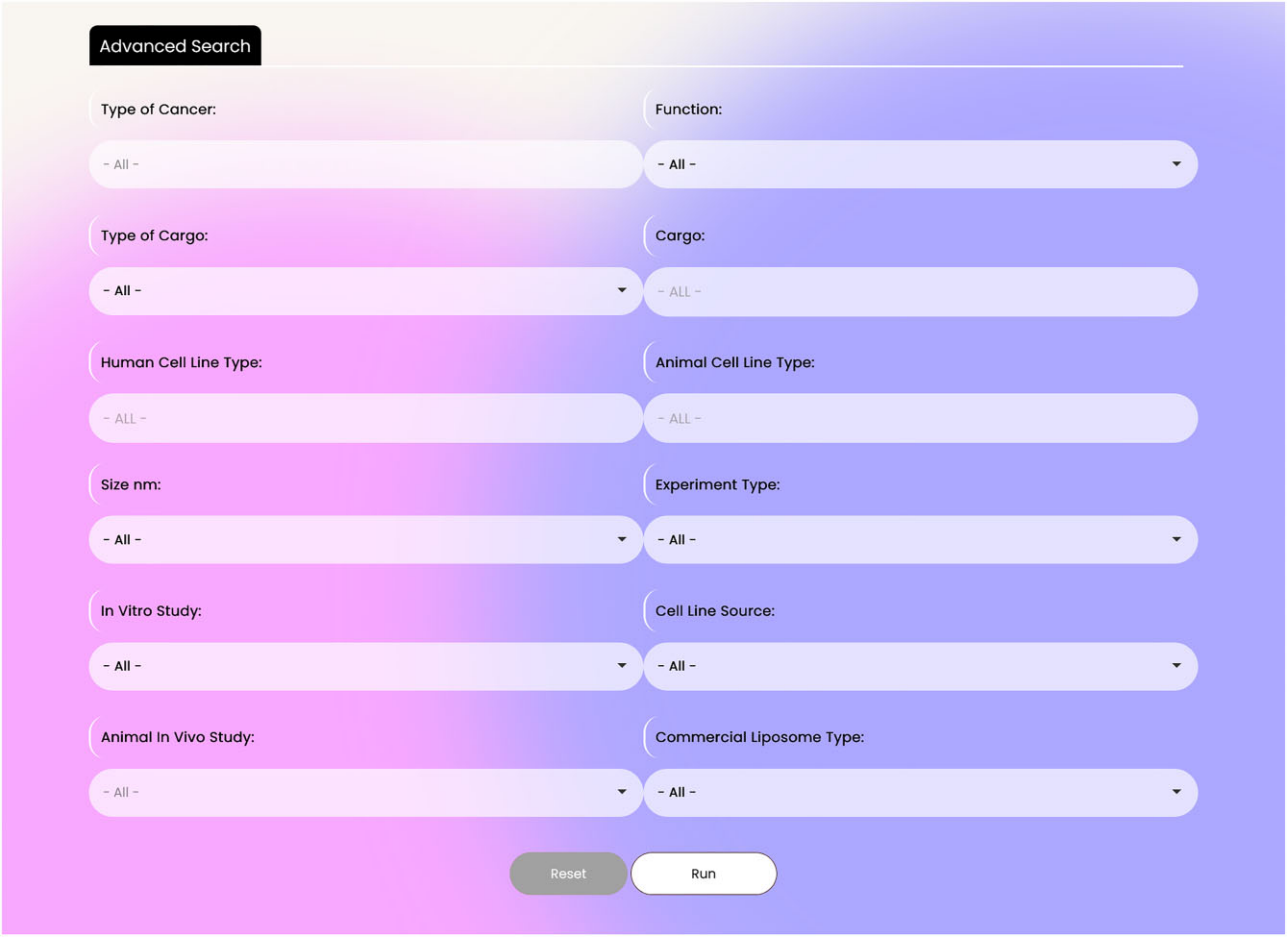
LCDD interface—advanced search.

The database contains two search parts: basic and advanced search. The database in its basic search has four search boxes containing: ‘Type of Cancer’, ‘Function’ (therapeutic, diagnostic, and theranostic), ‘Type of Cargo’ (nucleic acid, protein and peptide, chemical compounds, nanoparticles, lipid, and polysaccharides), and ‘Cargo’. These flexible search options help researchers to dig into the database easily (Fig. 2). The advanced search option, which is located in the page upper ribbon, provides eight more options rather than the four basic search options. The search items include: ‘Human Cell Line Type’, ‘Animal Cell Line Type’, ‘Liposome Size’, ‘Type of Experiment’, ‘Type of In Vitro Study’, ‘Cell Line Source’, ‘Animal In Vivo study’, and ‘Type of Commercial Liposomes’. In the ‘Type of Cancer’ section of the advanced search, multiples cancers can be selected simultaneously. A snapshot of the advanced search interface is presented in Fig. 3.

### Result section

**Figure 4.**
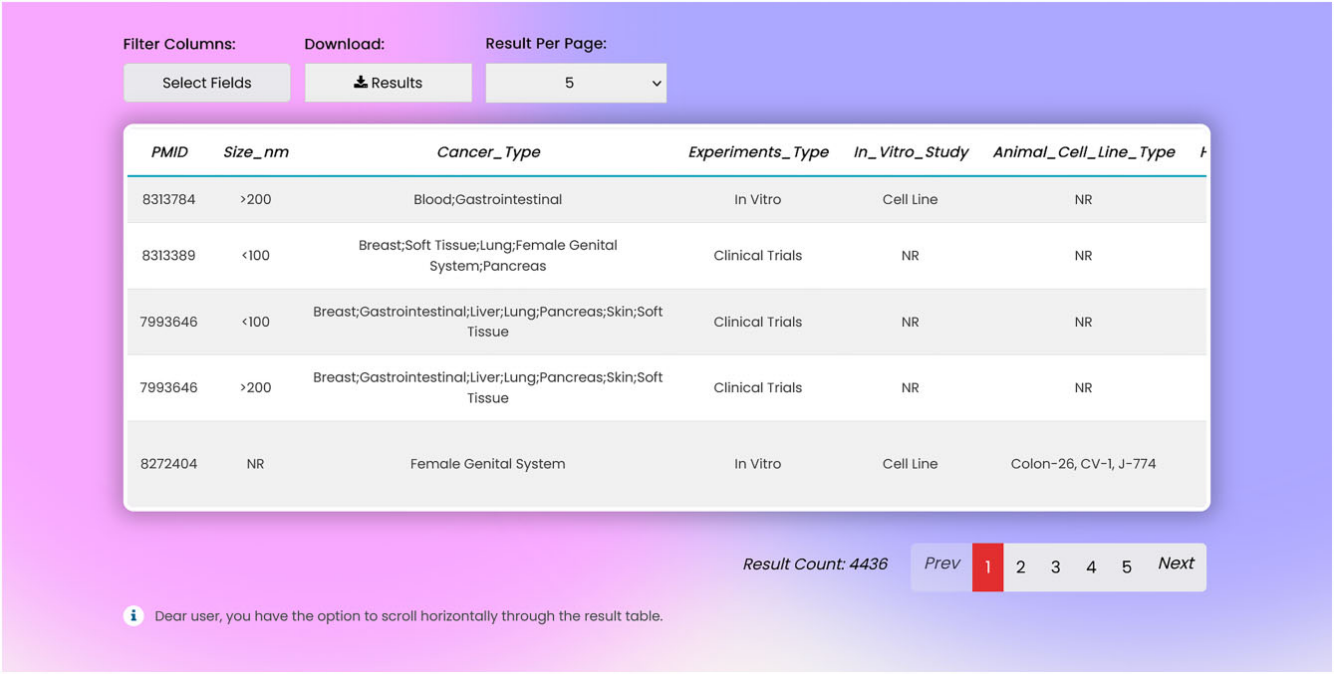
LCDD interface—search results.

In the result page, there is one row and several columns for each record. Columns include: PMID, liposome size, type of cancer, type of experiments, type of *in vitro* assays, type of cell line enrolled in the experiment, *in vivo* animal model, liposome function, loaded cargo, and type of commercial liposomes used in the study (Fig. 4). Entire or part of the data can be exported as .csv file from the search page.

**Figure 5.**
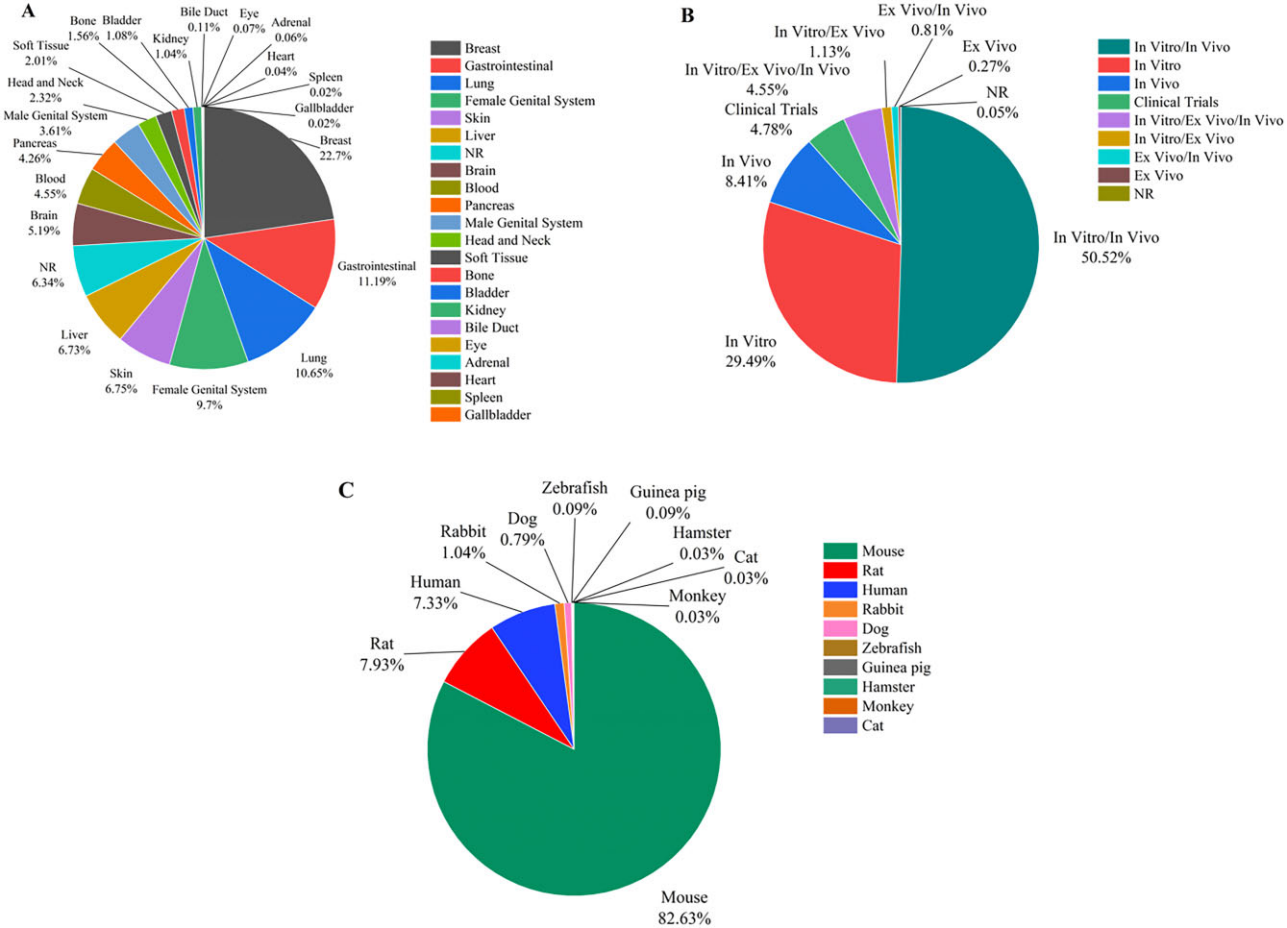
(A) The frequency of liposomal carriers used in various cancers, (B) the statistics of the experiment type in cancer-related liposomal formulations, and (C) the frequency of various animal models used in the *in vivo* experiment on liposomes in cancer-related research (NR denotes ‘not reported’).

By using various search combinations, users can not only sort different articles but also obtain various statistical results from the database. In Fig. 5A, the pie chart shows the frequency of liposomal nanoparticles used in different cancers studies. The five cancers most studied in liposome research are breast (22.7%), gastrointestinal (11.19%), lung (10.65%), female genital system (9.7%), and skin (6.75%) cancers. Gallbladder (0.02%), spleen (0.02%), and heart (0.04%) cancers are the least studied. As shown in Fig. 5B, *in vitro*/*in vivo* experiments (50.52%) and *in vitro* tests (29.49%) are the most common type of experiments that are used, while *ex vivo* studies are the least common, with about 0.27% of the studies. In the *in vivo* experiments of liposomal cancer drugs, mice (82.63%) were used in the majority of experiments, while monkeys, cats, and hamsters (all about 0.03%) were rarely used as animal models for liposomal cancer drug research (Fig. 5C). This can be attributed to the genomic similarity of mice to humans, ease of manipulation and handling, short life span, rapid reproduction, and established research protocols and cost-effectiveness compared to other lab animals [17, 18].

**Figure 6.**
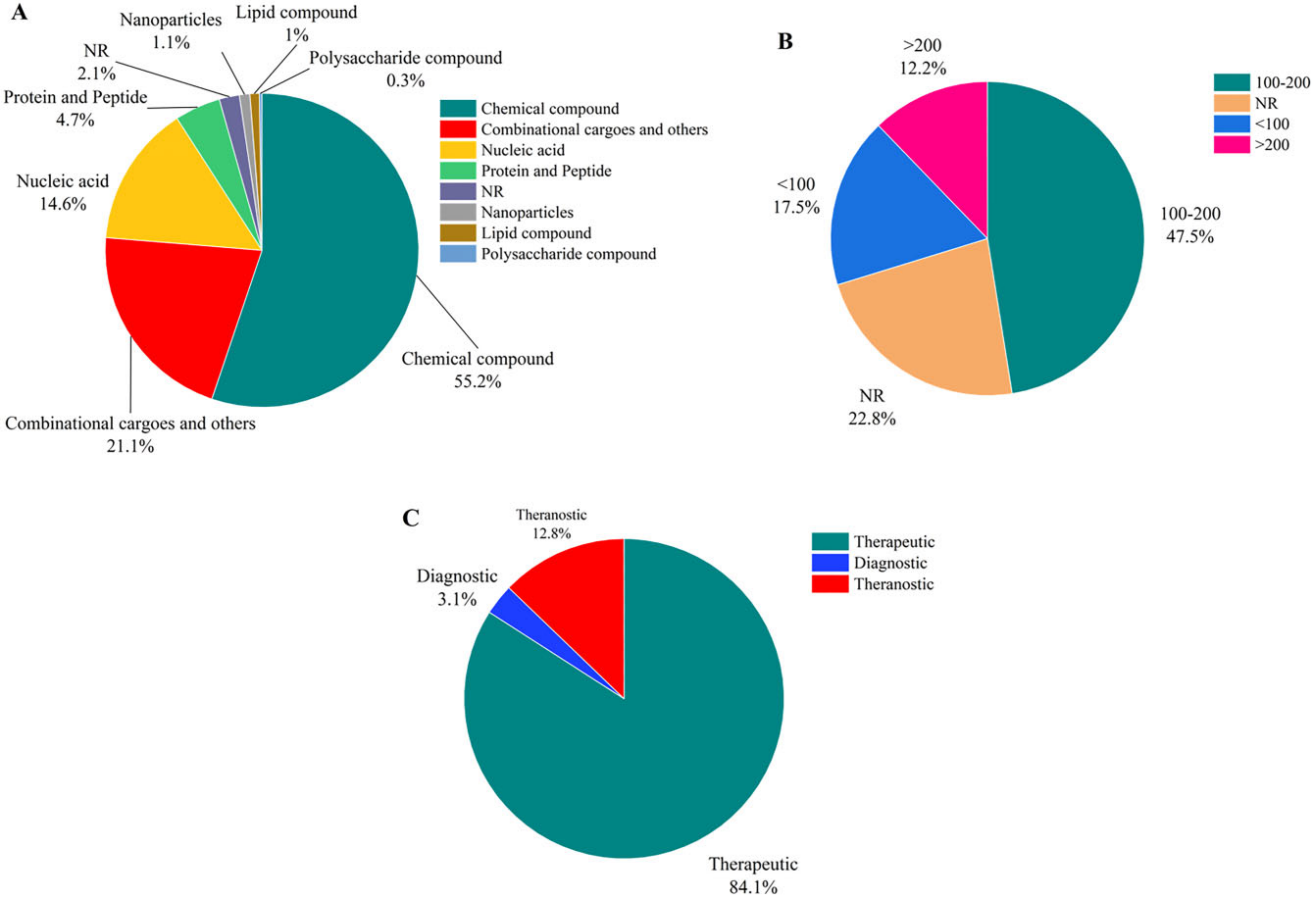
(A) The statistics of the cargos loaded in liposomal carriers, (B) the size distribution of the liposomal carriers in cancer research, and (C) statistics of therapeutic, diagnostic, and theranostic liposomal formulations (NR denotes ‘not reported’).

Figure 6A demonstrates that chemical compounds constitute about half of the cargos loaded on liposomes (55.2%). Nucleic acids (14.6%) and proteins (4.7%) are the second and third most commonly used cargos, respectively. However, combinational therapies by using two or more therapeutic agent are also increasingly being explored for cancer treatment (∼21.1%). These biological molecules can target specific genetic and molecular pathways involved in cancer development and progress, offering more precise and personalized treatment options. As presented in Fig. 6B, liposomes used as carriers for cancer drugs are mostly synthesized in 100–200 nm ranges (47.5%), while liposomes smaller than 100 nm and larger than 200 nm present in 17.5% and 12.2% of reported liposomes, respectively. As shown in Fig. 6C, liposomes are predominantly used for therapeutic purposes, accounting for 84.1% of all applications, while diagnostic purposes correspond to 3.1%. Theranostic applications account for 12.8% of liposomal carriers, highlighting the potential of liposomes in this area.

**Figure 7.**
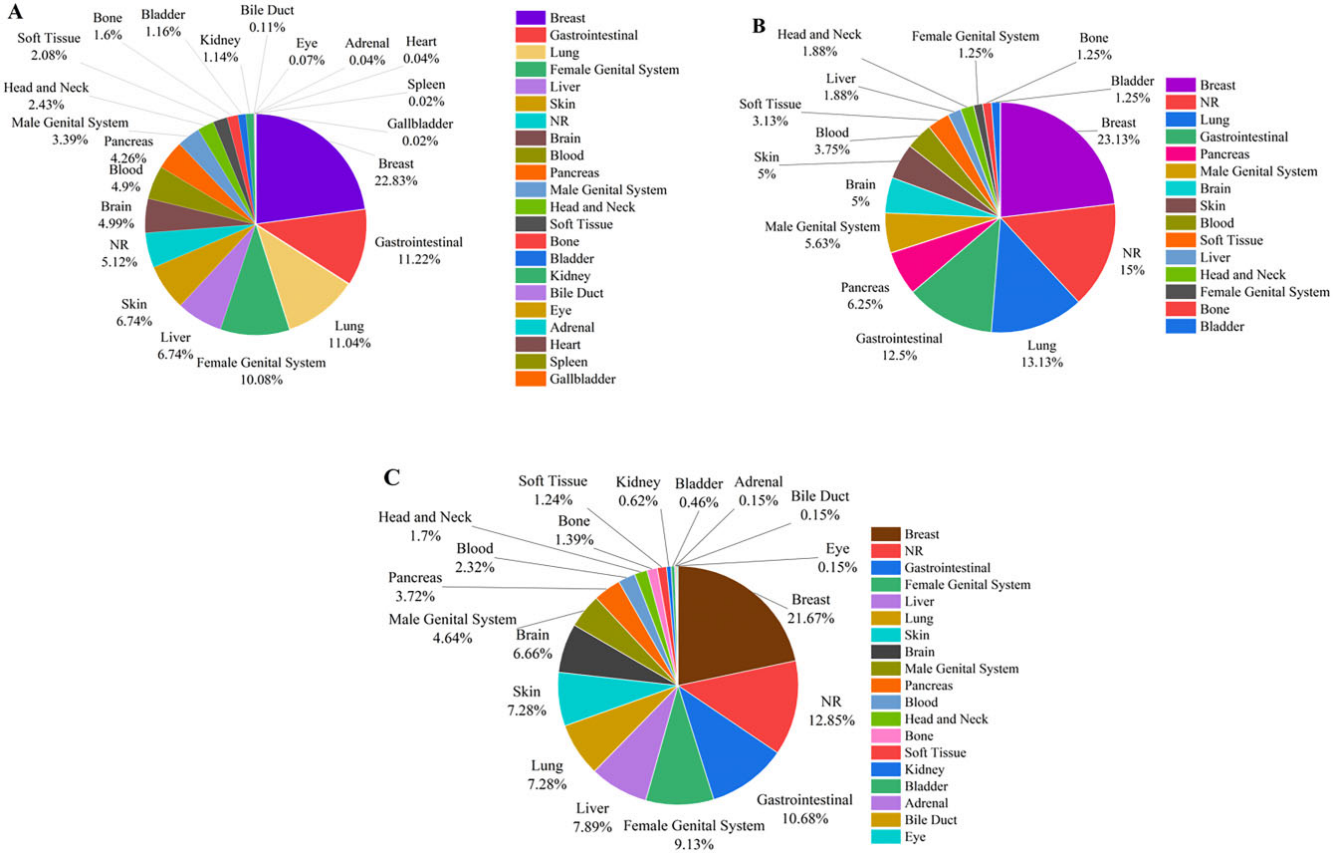
The statistics of using liposomal carriers for (A) therapeutic, (B) diagnostic, and (C) theranostic applications (NR denotes ‘not reported’).

Figure 7 demonstrates the statistics of therapeutic, diagnostic, and theranostic liposomal carriers in various cancers. As shown in Fig. 7A, breast (22.83%), gastrointestinal (11.22%), and lung (11.04%) cancers are the top studied cancers in therapeutic liposomes. Diagnostic liposomal carriers have been mostly used for breast (23.13%), lung (13.13%), and gastrointestinal (12.5%) cancers (Fig. 7B). As represented in Fig. 7C, breast (21.67%), gastrointestinal (10.68%), and female genital system (9.13%) are among the top cancers for the theranostic liposomes.

**Figure 8.**
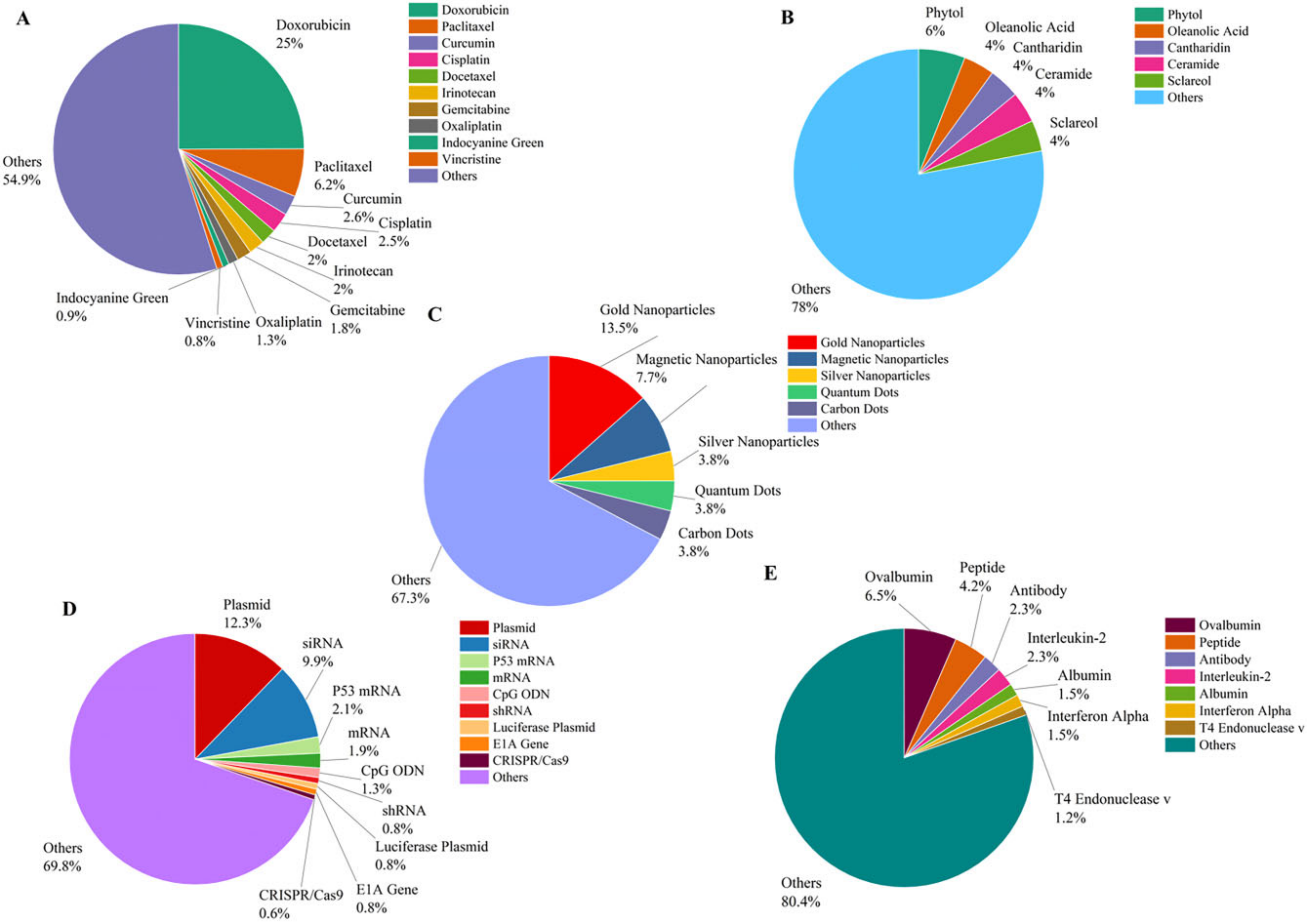
The statistics of the most frequently used cargoes based on their types, including (A) chemical compounds, (B) lipid compounds, (C) nanoparticles, (D) nucleic acids, and (E) proteins and peptides.

Figure 8 summarizes the most used drugs/cargoes in each category of cargoes. Doxorubicin is the most used cargo among the chemical compounds (Fig. 8A). Phytol is the mostly loaded lipid-based cargo in the liposomes (Fig. 8B). Gold nanoparticles, which are also the most widely used nanoparticles in biomedicine, constitute the largest part in the nanoparticle category (Fig. 8C). Among various nucleic acids, plasmids and siRNA are the most loaded cargoes on liposomes (Fig. 8D). Ovalbumin as the most studied cargo in the protein and peptide category is a protein antigen widely used in cancer research, particularly in immunotherapy and vaccine development (Fig. 8E).

Analysing different cell lines in liposome-based cancer drugs, 4T1 (murine breast cancer cell line), B16-F10 (murine melanoma cell line), and CT2 (murine colon carcinoma cell line) with 250, 125, and 54 studies are the most three studied animal cell lines. MCF7 (breast cancer cell line), A549 (lung cancer cell line), and MDA-MB-231 (breast cancer cell line) with 434, 186, and 171 studies are the most three studied human cell lines in the liposome studies.

### Advantages and the expanding scope of LCDD

To the best of our knowledge, no other database is specifically dedicated to liposomal anticancer studies. While researchers can utilize PubMed or Scopus to find related articles, the LCDD offers a more targeted, goal-oriented, and user-friendly experience. It enhances efficiency by saving time, improving accuracy, and enabling seamless study comparisons. Furthermore, LCDD acts as a plug-and-play resource for computational research, including machine learning, trend analysis, and meta-reviews. Users are encouraged to use the technical support tab to explore potential collaborations involving data analysis and computational research. Although high-quality review articles exist on liposomal anticancer therapeutics, none provide the same level of depth and structured organization as LCDD. Additionally, LCDD’s regular updates every 6 months ensure coverage of the latest research, surpassing the static nature of published reviews.

However, lack of access to full-text articles is a limitation of this database. To help users locate original articles, PMID numbers have been provided. A ‘contact us’ tab is available for users to report typos or bugs.

## Conclusion and perspectives

Liposomes make up the majority of clinically approved nanocarriers for anticancer agents. They are highly regarded for their favourable attributes, offering numerous opportunities for diagnosis and treatment of a variety of cancers. In this study, we manually extracted data from 4436 research articles on the development and characterization of therapeutic, diagnostic, and theranostic liposomes for cancer diagnosis and treatment. This data have been compiled into a comprehensive database, LCDD. The data were checked multiple times to ensure reliability, making it a valuable reference for those using liposome formulations in cancer therapy or diagnosis. LCDD offers several benefits, including centralized information, enhanced collaboration, and improved efficiency by avoiding duplication of efforts. It provides global accessibility, ensuring that researchers worldwide can benefit from shared knowledge. LCDD serves as an educational tool, allows for analysis of trends and identification of research gaps. Data extracted from the LCDD show that breast cancer is the most studied cancer type in liposome research, followed by gastrointestinal, lung, and female genital system cancers. LCDD showed that liposomes are mostly studied for therapeutic rather than diagnostic applications. Moreover, analysing statistics of drugs used in liposomal anticancer formulations, revealed trends linking database outputs, clinical trials, and marketed drugs. Chemical compounds, especially doxorubicin, dominate the most frequently loaded cargoes in liposomes across research, clinical trials, and the market. While anticancer therapeutic miRNAs are gaining attention, they are less studied, marketed, or trialled compared to chemical compounds (Tables 1 and 2). Several factors, such as miRNA instability in the body, off-target effects, and immunogenicity could hamper the clinical application of miRNA. These findings may inspire research in underexplored areas and bridge the gap to market-ready innovations.

**Table 1.** Liposomal products in clinical trial phases yet to be approved with their intended clinical application (not exhaustive) (obtained from the https://clinicaltrials.gov/) [19]

Product name	Drug	Cancers	Status
L-NDDP/Aroplatin™	*cis*-*bis*-Neodecanoato-*trans*-R,R-1,2-diaminocyclohexane platinum	B-cell lymphoma, malignant mesothelioma, pancreatic cancer, colorectal cancer, solid tumours	Phase I/II
BP1002 (US)	Antisense oligonucleotide against BCl-2	Acute myeloid leukemia, advanced lymphoid malignancies	Phase I
EndoTAG^®^ (EU, US, TW, UA)	Paclitaxel	Breast cancer, pancreatic cancer, liver cancer	Phase II/III
PLM60 (US, CN)	Mitoxantrone	Advanced hepatocellular carcinoma, small-cell lung cancer, non-Hodgkin’s lymphoma, recurrent/refractory lymphomas	Phase I/II
ThermoDox^®^ (US)	Doxorubicin	Hepatocellular carcinoma, colorectal cancer, pediatric cancer, liver neoplasms, pancreatic cancer, breast cancer	Phase I/II/III
LiPlaCis (DK)	Cisplatin	Adv./refractory solid tumours, metastatic breast cancer, prostate cancer, skin cancer	Phase I/II
Lipoplatin™	Cisplatin	Malignant pleural effusions	Phase I
SPI-077	Cisplatin	Ovarian cancer	Phase II
R-CMOP	Mitoxantrone hydrochloride, cyclophosphamide, vincristine, prednisone, and rituximab	Diffuse large B-cell lymphoma	phase I/II
Liposomal Bupivacaine	Bupivacaine	Pain control following pancreatoduodenectomy	Phase II
Pembrolizumab	Pembrolizumab	Classical Hodgkin lymphoma	Phase II
Adjuvant Liposomal Doxorubicin	Doxorubicin	Early-stage triple-negative breast cancer	Phase II
Mitoxantrone Hydrochloride Liposome	Mitoxantrone hydrochloride	Relapsed/​refractory diffuse large B-cell lymphoma (DLBCL)	Phase II
Liposomal Doxorubicin	Doxorubicin	Newly diagnosed glioblastoma	Phase II
Liposomal Paclitaxel	Paclitaxel	Locally advanced non-small cell lung cancer	Phase II
Doxil	Doxorubicin	Metastatic breast cancer (HER2+)	Phase II
Liposomal Doxorubicin	Doxorubicin	Advanced sarcoma	Phase I
Liposomal Mitoxantrone	Mitoxantrone	Newly diagnosed acute myeloid leukemia (AML)	Phase II
Pegylated Liposomal Doxorubicin	Doxorubicin	Ovarian cancer	Phase I
BP1001	Grb2 antisense oligonucleotide, venetoclax, and decitabine	Refractory/relapsed AML	Phase II

**Table 2.** Approved liposomal products for chemotherapy with their year of approval, intended clinical application, and their marketing status [19]

Product name	Drug	Indication	Approval date	Marketing status
DaunoXome^®^ (US)	Daunorubicin	AIDS-related Kaposi’s sarcoma	1995–1997 (EMA) 1996 (FDA)	US discontinued (2016), global on-demand access (2016, EU, UK, AU, NZ, HK)
DepoCyt^®^ (US, EU)	Cytarabine	Lymphomatous meningitis	1999 (FDA) 2001 (EMA)	Withdrawn production by company (2017)
Doxil^®^ (US)/Caelyx^®^ (EU)^a^	Doxorubicin	AIDS-related Kaposi’s sarcoma, recurrent ovarian cancer, multiple myeloma, metastatic breast cancer (EU only)	1995 (FDA) 1996 (EMA)	Active (US, EU)
Lipo-Dox^®^ (TW)	Doxorubicin	AIDS-related Kaposi’s sarcoma, ovarian cancer, breast cancer, multiple myeloma	1998 (TW)	Active (TW)
Marqibo^®^ (US)	Vincristine	Acute lymphoblastic leukemia	2012 (FDA)	US discontinued (2020)
Mepact^®^ (EU)	Mifamurtide	Osteosarcoma	2009 (EMA)	Active (EU)
Myocet^®^ (EU)	Doxorubicin	Metastatic breast cancer	2000 (EMA)	Active (EU)
Onivyde^®^/Nal-IRI (EU, US)	Irinotecan	Pancreatic cancer	1996 (FDA) 2016 (EMA)	Active (US, EU)
Vyxeos^®^/CPX-351 (EU, US)	Cytarabine: daunorubicin (5:1 mol. ratio)	Newly diagnosed therapy-related acute myeloid leukemia, acute myeloid leukemia with myelodysplasia-related changes	2017 (FDA) 2018 (EMA)	Active (US, EU)
Zolsketil^®^ (EU)	Doxorubicin	Metastatic breast cancer, advanced ovarian cancer, multiple myeloma, AIDS-related Kaposi’s sarcoma	2022 (EMA)	Active (EU)

a Bioequivalent.

LCDD provides detailed information about various liposomal formulations, which will be helpful for big data analysis and assist researchers in making better decisions in conducting their research on liposomal preparations for cancer therapy and diagnosis. Pharmaceutical companies and researchers can use the database to identify promising liposomal formulations and streamline the drug development process. It serves as a valuable resource for students and educators, providing access to a wealth of information for learning and teaching purposes. Authors are dedicated to maintaining and updating the LCDD, addressing bugs, developing comprehensive documentation, creating user-friendly tutorials, and enhancing LCDD functionalities. Additionally, to improve user experience and search relevance, ontology-based search integration will be explored. Users are encourage to reach authors at https://bioinformaticscollege.ir/lcdd/contact-us/ for bug report, requests for assistance, and suggestions for new features and updates. Feedbacks from all users are welcomed to continuously improve the platform.

## Data Availability

The LCDD database will be updated regularly (every 6 months). It is now publicly and freely available at https://bioinformaticscollege.ir/lcdd/. Related data will be available through request from the authors.
